# Multi-drug resistant *Acinetobacter *infections in critically injured Canadian forces soldiers

**DOI:** 10.1186/1471-2334-7-95

**Published:** 2007-08-14

**Authors:** Homer C Tien, Anthony Battad, Elizabeth A Bryce, Jeffrey Fuller, Michael Mulvey, Kathy Bernard, Ronald Brisebois, Jay J Doucet, Sandro B Rizoli, Robert Fowler, Andrew Simor

**Affiliations:** 1 The Trauma Program, and the Department of Surgery, Sunnybrook Health Sciences Centre, H186-2075 Bayview Avenue, Toronto, Canada, M4N 3M5; 2 1st Canadian Field Hospital, CFB Petawawa, Petawawa, Ontario, Canada; 3 Department of Pathology and Laboratory Medicine, Vancouver General Hospital, Vancouver, Canada; 4 Department of Laboratory Medicine and Pathology, University of Alberta Hospital, Edmonton, Canada; 5 Nosocomial Infections and Antimicrobial Resistance Laboratory, National Microbiology Laboratory, Winnipeg, Canada; 6 Departments of Surgery and Critical Care Medicine, University of Alberta Hospital, Edmonton, Canada; 7 Department of Surgery, Vancouver General Hospital, Vancouver, Canada; 8 Department of Critical Care Medicine, Sunnybrook Health Sciences Centre, Toronto, Canada; 9 Department of Microbiology, Sunnybrook Health Sciences Centre, Toronto, Canada

## Abstract

**Background:**

Military members, injured in Afghanistan or Iraq, have returned home with multi-drug resistant *Acinetobacter baumannii *infections. The source of these infections is unknown.

**Methods:**

Retrospective study of all Canadian soldiers who were injured in Afghanistan and who required mechanical ventilation from January 1 2006 to September 1 2006. Patients who developed *A. baumannii *ventilator associated pneumonia (VAP) were identified. All *A. baumannii *isolates were retrieved for study patients and compared with *A. baumannii *isolates from environmental sources from the Kandahar military hospital using pulsed-field gel electrophoresis (PFGE).

**Results:**

During the study period, six Canadian Forces (CF) soldiers were injured in Afghanistan, required mechanical ventilation and were repatriated to Canadian hospitals. Four of these patients developed *A. baumannii *VAP. *A. baumannii *was also isolated from one environmental source in Kandahar – a ventilator air intake filter. Patient isolates were genetically indistinguishable from each other and from the isolates cultured from the ventilator filter. These isolates were resistant to numerous classes of antimicrobials including the carbapenems.

**Conclusion:**

These results suggest that the source of *A. baumannii *infection for these four patients was an environmental source in the military field hospital in Kandahar. A causal linkage, however, was not established with the ventilator. This study suggests that infection control efforts and further research should be focused on the military field hospital environment to prevent further multi-drug resistant *A. baumannii *infections in injured soldiers.

## Background

*Acinetobacter baumannii *is a gram-negative bacterium that can cause infectious outbreaks in critically-ill patients, often with limited treatment options due to antibiotic resistance [1-3]. In November, 2004, the Centers for Disease Control and Prevention (CDC) reported an increasing number of multi-drug resistant (MDR) *A. baumannii *bloodstream infections in United States military personnel returning from service in Iraq and Afghanistan [4]. The source of these infections is unknown. We report the results of an infection control investigation of four Canadian Forces members who developed ventilator associated pneumonia (VAP) from MDR *A. baumannii *after injury in Afghanistan.

## Methods

In November 2005, Canadian Forces (CF) members deployed to Kandahar, Afghanistan. Surgical support was provided by the 248^th ^Combat Support Hospital, a US medical unit. However, a Canadian-led medical unit (Role Three Multinational Medical Unit (R3MMU)) assumed command of the facility on February 7, 2006. Two of the authors (HCT and AB) were part of the first Canadian medical contingent to arrive at this facility in Kandahar. Three mechanical ventilators (iVent201, Versamed Inc, Pearl River, NY) were purchased from the departing US organization, and were left in the facility for use by the R3MMU. At the time of the hand-over, this facility had 11 beds in a small, common room. The intensive care unit consisted of three of these beds which were equipped with the iVent ventilators. The R3MMU has basic laboratory support, but does not have any microbiological testing capability.

While working at this hospital, the authors (HT and AB) treated a Canadian soldier (patient one) who suffered a severe head injury and required mechanical ventilation. After this patient was evacuated back to Canada, they were informed that he developed MDR *A. baumannii *VAP. As a result, they took environmental samples from inside and around the hospital. These included numerous soil samples from around the operating room, ICU and resuscitation bay. Because the hospital did not have microbiological testing capabilities, they collected all samples using sterile forceps or sterile basins, and placed them into sterile specimen containers. Also, they dismantled the patient's (patient one) mechanical ventilator, and collected samples (including the air intake filter) from it using sterile forceps and specimen containers. These were all couriered to microbiology laboratories in Canadian hospitals for testing. The samples arrived in Canada approximately 72 hours after collection.

Several months after returning to Canada, the authors(HCT & AB) then identified all CF members who were injured in Kandahar and who required mechanical ventilation using aeromedical evacuation records (obtained through the Canadian Forces Health Services) from 1 January 2006 to 1 September 2007. In general, injured CF members are evacuated by US Air Force medical teams to a US army hospital in Germany (Landstuhl Regional Medical Center or LRMC). Canadian medical teams then bring casualties from LRMC to civilian Canadian hospitals. During the study period time, CF members were repatriated to five different civilian tertiary care centers in Canada.

We reviewed the medical records of these patients from Kandahar, LRMC and their respective Canadian hospitals, if available. We also contacted the attending physicians responsible for each patient in Canada to discuss each patient's clinical course in hospital. We also contacted the microbiology department of LRMC and all involved Canadian hospitals to obtain the microbiological record for each patient. Our primary outcome of interest was the development of *A. baumannii *ventilator associated pneumonia, as defined by the CDC's National Nosocomial Infection Surveillance system [5]. *A. baumannii *respiratory isolates were then retrieved, if available, for all patients who developed *A. baumannii *VAP. Patient isolates were then compared to each other, and to any positive environmental isolates from the hospital in Kandahar.

Organisms were identified using standard laboratory methods, including Gram stain appearance, colonial morphology, negative oxidase reaction, and the VITEK-2 ID-GNB card (bioMérieux, Hazelwood, MO). Antibiotic susceptibility testing was done using the VITEK-2 AST-N019 card according to the manufacturer's instructions (bioMérieux). Genomic DNA from all *A. baumannii *isolates was digested with *Apa*I for molecular typing by PFGE [6]. Interpretation of DNA profiles generated by PFGE was done visually. Isolates were considered to be genetically related if their PFGE patterns differed by three of fewer bands [7].

Institutional review ethics approval was obtained to conduct this study. However, individual consent was not obtained from each patient as some had been already discharged from hospital. As the number of critically wounded CF casualties was small during the study period, we only present aggregate demographic data (means ± standard deviation), as individual information may identify specific patients. Statistical analysis was performed using SAS version 8.02 (SAS Institute, Carey, NC).

## Results

During the study period, six CF members required mechanical ventilation and were evacuated through LRMC to five different Canadian hospitals. Four of these patients developed multi-drug resistant *A. baumannii *VAP. Their mean age was 26.5 years (± 8.5). Three sustained multiple injuries from improvised explosive devices; one received a severe head injury. On average, they required 68 days of mechanical ventilation (± 70 days).

During this same period of time, a total of 42 injured CF members were evacuated from Kandahar to Canadian hospitals, through Landstuhl Regional Medical Centre. On review of the *A. baumannii *screening results from LRMC (which was performed shortly after admission), there were skin culture reports found for 25 of these patients; reports were not found for 17. Only two patients had positive screening tests that were positive for *A. baumannii *(preliminary, non-published results). One of the positive screening tests was from one of the four study patients (patient one). Unfortunately, the isolate was not available for testing by PFGE.

**Figure 1:**
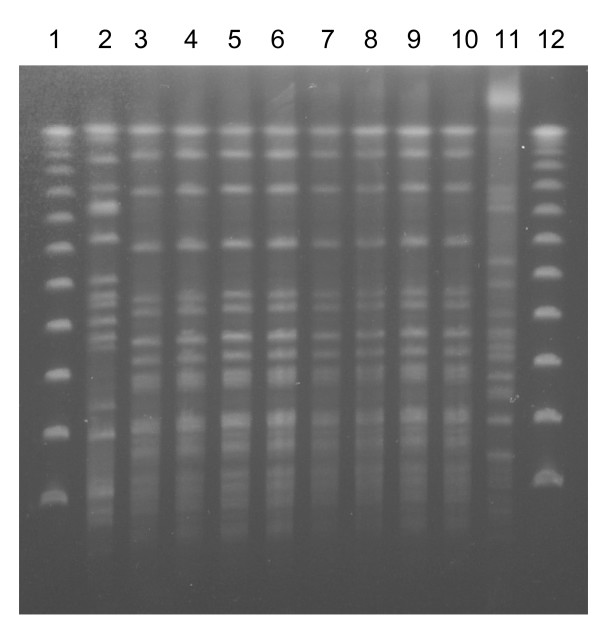
**DNA fingerprint profiles of *Acinetobacter baumannii *after *Apa*I-digested pulsed-field gel electrophoresis**. Lanes 1 and 12: Lambda DNA ladder (New England Biolabs, Mississauga, ON) Lanes 2 and 11: Unrelated *A. baumannii *strains Lane 3: Patient 1 (bronchoalveolar lavage (BAL) from LRMC) Lane 4: Patient 1 (Tracheal aspirate, Canada) Lane 5: Patient 2 (Tracheal aspirate, LRMC) Lane 6: Patient 2 (Tracheal aspirate, Canada) Lane 7: Patient 3 (Tracheal aspirate, LRMC) Lane 8: Patient 3 (BAL, Canada) Lane 9: Patient 4 (BAL, Canada) Lane 10: *A. baumannii *isolate from air intake filter, Kandahar.

The *A. baumannii *respiratory isolates retrieved from Canadian hospitals and from LRMC for these four patients were molecularly identical based on the PFGE (See Figure 1). For patient one, a multi-drug resistant strain of *A. baumannii *was cultured from a bronchoalveolar lavage (BAL) specimen obtained five days post injury at LRMC. The BAL was done for signs and symptoms suggesting VAP, including a fever and an infiltrate on chest radiography. This strain of *A. baumannii *persisted in respiratory samples isolated from the Canadian hospital. The *A. baumannii *isolates for patients two and three, obtained from LRMC on post-injury days ten and twelve respectively were multi-drug resistant and identical to the multi-drug resistant strain from patient one (Figure 1). Both patients were showing signs of VAP (fever and infiltrates on chest radiography) at the time of these positive cultures. These same strains were also cultured from respiratory samples from patients two and three in their respective Canadian hospitals. *A. baumannii *respiratory isolates from patient four, cultured two weeks post injury in Canada were also identical to this multi-drug resistant strain (Figure 1). Patient four also had clinical and radiological signs of VAP, at the time of this positive result. Respiratory isolates from LRMC for patient four were negative for *A. baumannii*.

*A. baumannii *was not isolated from any environmental samples, except from one ventilator air intake filter sample from Kandahar. This filter isolate was also multi-drug resistant, and identical to the MDR strain obtained from all four patients in LRMC and Canada. The antibiogram for the *A. baumannii *strains isolated from all four patients at their various hospitals showed identical antibiotic susceptibilities (See Table 1).

**Table 1:** Antibiogram of MDR *A. baumanni *isolates

**Antibiotic**	**Sensitivity (MIC) MDR *A. baumannii *isolates**
Ampicillin	R (≥ 32)
Gentamicin	R (≥ 16)
Trimethoprin/Sulfa	R (≥ 16)
Cefotaxime	R (≥ 64)
Ciprofloxacin	R (≥ 4)
Cefazolin	R (≥ 64)
Ampicillin/Sulbactam	R (≥ 32/16)
Amikacin	**S **(≤ 2)
Ceftazidime	R (≥ 64)
Cefuroxime	R (≥ 64)
Imipenem	R (≥ 16)
Mezlocillin	R (≥ 128)
Piperacillin	R (≥ 128)
Tetracycline	R (≥ 16)
Tobramycin	**S **(≤ 1)
Cefepime	R (≥ 64)
Levofloxacin	R (≥ 8)

MIC, Mean inhibitory concentration (mcg/mL);

R, Resistant;

S, Sensitive;

## Discussion

We report four cases of multi-drug resistant *A. baumannii *infections in CF members injured in Afghanistan. MDR *A. baumannii *VAP was first diagnosed in three of these four patients at the US army hospital in Landstuhl, Germany and continued to be an issue for them in Canada. Patient four developed *A. baumannii *VAP while hospitalized in Canada. Originally, we suspected that the source of these infections was LRMC, as all four patients had molecularly identical strains of MDR *A. baumannii *VAP, and all four had been treated at LRMC where MDR *A. baumannii *infections are prevalent. However, the filter isolate was also identical to the MDR *A. baumannii *isolated from these four patients, suggesting that the field hospital was the original source of infection.

We were only able to demonstrate *A. baumannii *in one environmental sample – the ventilator filter isolate. If the field hospital were contaminated, we would expect fairly widespread contamination and therefore, would have expected multiple environmental samples to have been positive for *A. baumannii*. However, as mentioned previously, the R3MMU field hospital does not have microbiological testing capability; therefore, all samples were couriered to Canada for culture. This resulted in a delay of at least 72 hours prior to processing; this delay may be responsible for the low yield of positive cultures in our environmental samples.

The origin of the *A. baumannii *infections in soldiers injured in Afghanistan and Iraq is still unknown. These infections may have been acquired from environmental sources in the field, during treatment at military field hospitals, during evacuation from the theatre of operations, or during treatment at hospitals outside of the conflict area. During the Vietnam War, *A. baumannii *was recovered from traumatic injuries to extremities [8]. More recent reports have also identified *A. baumannii *infections in patients who suffered traumatic injuries, suggesting environmental contamination of wounds as a potential source [9]. Our report also suggests that these MDR *A. baumannii *organisms originated from an environmental source in the theatre of operations. All MDR *A. baumannii *isolates from these four patients were molecularly identical to the ventilator filter isolate from Kandahar.

*A. baumannii *has often been linked to infectious outbreaks involving the mechanical ventilation circuit [10-12]. However, this study does not definitively establish a causal link between the contaminated ventilator in Kandahar and the subsequent four cases of MDR *A. baumannii *VAP. *A. baumannii *is a ubiquitous micro-organism that colonizes inanimate surfaces and medical equipment. Our patients may have just as easily acquired the MDR *A. baumannii *through lack of hand hygiene in health workers, microaspiration or even hematogenous spread from an infected IV site. Once acquired, the ventilator may have been secondarily contaminated.

Unfortunately, because of limitations of the field environment, we were unable to trace contact exposures of infected patients back to specific health care workers. Because of limitations in personnel, patients were not assigned to specific health care workers; instead, two to three nurses were on duty for each shift, and collectively cared for all patients in the facility. As a result, all patients were exposed to all health care workers because of the small numbers of hospital staff. Likewise, we were unable to screen the hands of hospital health care workers, as the facility did not have microbiological testing capability.

Even so, the results of this report suggest that the field hospital environment is the source of MDR *A. baumannii *infections and that increased infection control measures are required at the field hospital level in military theatre of operations. For example, regular ventilator cleaning and maintenance is difficult in the field setting, and outgoing staff from the 248^th ^CSH reported that air-intake filters and antibacterial circuit filters were difficult to obtain. As a result, antibacterial circuit filters were not routinely used, and the air-intake filters were not routinely changed. Based on our findings, the ventilators are now regularly maintained, and air-intake filters are changed as recommended by the manufacturer. Anti-bacterial circuit filters are also now used (Intersurgical Filta-Therm Filter, Intersurgical Ltd, Wokingham Berkshire UK). As well, hospital cleaners have been hired and hand-washing stations have been established in patient care areas.

Our findings are likely applicable to US and British military field hospitals as well. Our ventilators were previously used by the 248^th ^CSH. As well, the environmental and logistical problems that faced both the 248^th ^CSH and the R3MMU are common across field hospitals in Afghanistan and Iraq [13,14]. Other contributory factors may have included non-restrictive antibiotic use at the field hospitals to compensate for poor environmental conditions [13].

Identifying the etiology of multi-drug resistant *Acinetobacter *infection in returning soldiers is important for preventing nosocomial transmission to other vulnerable patients. Zapor reported 53 cases of nosocomial transmission of MDR *Acinetobacter *that have directly resulted in four fatalities in US military hospitals [15]. Jones has also reported on one British service member who may have imported an MDR *A*. *baumannii *strain from Iraq that caused a large outbreak in National Health Service hospitals in the UK [14].

Ongoing surveillance remains critical for monitoring the efficacy of infection control measures. In Canada, critically injured service members are cared for in the civilian health care system. As a result, the Canadian Forces Health Services is collaborating with the Public Health Agency of Canada (PHAC) to implement a national surveillance strategy for MDR *Acinetobacter *infections in injured soldiers. The multinational nature of military coalitions and medical care, however, highlight the need for health care providers to also communicate across national borders.

## Conclusion

We conclude that the military field hospital was the source of multi-drug resistant *A. baumannii *that later lead to the development of ventilator associated pneumonia in four CF members who were injured in Kandahar, Afghanistan. Further research and infection control efforts should be focused on the field hospital environment to prevent future *A. baumannii *infections.

## Competing interests

HCNT, AB, RB, JD are serving medical officers in the Canadian Forces Health Services.

## Authors' contributions

HCNT and AB gathered all environmental samples in Kandahar. AS, JF, EAB and JD provided patient isolates from Canadian hospitals. HCNT and JD facilitated the ethics review process for this project. HCNT, RB and JD obtained clinical information concerning the clinical course of each patient. The labs of MM and AS performed PFGE on environmental samples from Kandahar. The lab of AS performed PFGE on all patient isolates from all hospitals. MM, KB, AS, RB, SR provided methodological advice and background information on this project. HCNT wrote the manuscript. All authors read and approved the final manuscript.

## Pre-publication history

The pre-publication history for this paper can be accessed here:


